# Balancing diagnostic approach and oncologic risk in surgically resected lung cancer: propensity-matched analysis of intraoperative versus percutaneous biopsy by tumor location and morphology

**DOI:** 10.1097/JS9.0000000000005106

**Published:** 2026-03-17

**Authors:** Younggi Jung, Eunjue Yi, Sung Ho Hwang, Sungho Lee, Jae Ho Chung

**Affiliations:** a Department of Thoracic and Cardiovascular Surgery, Korea University Anam Hospital, Korea University College of Medicine, Seoul, Republic of Korea; b Department of Radiology, Korea University Anam Hospital, Korea University College of Medicine, Seoul, Republic of Korea

**Keywords:** biopsy, needle/biopsy, surgical/lung cancer/propensity score/recurrence

## Abstract

**Background:**

Lung cancer remains the leading cause of cancer-related death, and earlier detection has increased the demand for accurate histological diagnosis While percutaneous transthoracic needle biopsy (PCNB) and surgical biopsy are widely used, concerns remain about PCNB-related tumor dissemination and recurrence. These risks are influenced by tumor location and morphology, which affect procedural feasibility and oncologic outcomes. This study investigated the association between biopsy method and recurrence-free survival (RFS) in surgically resected lung cancer and evaluated how tumor characteristics may identify subgroups at higher risk.

**Materials and Methods:**

Medical records of 363 patients with surgically resected primary lung cancer who underwent preoperative PCNB (*n* = 221) or intraoperative surgical biopsy (*n* = 142) at a tertiary center between 2015 and 2020 were retrospectively reviewed. Patients were grouped according to biopsy methods and compared. Demographics, preoperative chest CT findings such as consolidation to tumor ratio (CTR) and intralobar tumor location, pathologic findings, and recurrences were evaluated.

**Results:**

In the propensity-matched cohort (103 patients per group), multivariate analysis showed male (HR 2.11), current smoking (HR 2.12), central tumors (HR 5.47), and higher CTR (HR 16.6) as independent predictors of reduced RFS, whereas PCNB itself was not (*P* = 0.16). CTR and tumor location remained important risk factors in both groups. Notably, locoregional and pleural recurrences were more frequent in the PCNB group, although not statistically significant. Five-year RFS was significantly lower in the PCNB group (85.0% vs. 74.3%, *P* = 0.013), with the negative impact of higher CTR and central tumors being more pronounced in the PCNB group.

**Conclusion:**

PCNB was not an independent risk factor for recurrence. However, its association with higher rates of locoregional and pleural recurrence along with a trend toward decreased RFS in tumors with adverse CT features suggests careful clinical consideration when choosing biopsy method.

## Introduction

Lung cancer is the most prevalent cancer and the leading cause of cancer-related mortality worldwide, with its increasing incidence linked to advancements and wider utilization of screening methods^[^1^]^. Among these, low-dose computed tomography (LDCT) has markedly improved the early detection of lung cancer and contributed to reduced mortality, as demonstrated in key landmark randomized trials such as the National Lung Screening Trial (NLST) and NELSON trial^[^2,3^]^. These findings established LDCT as the standard screening method for lung cancer.

With the increased detection of nodules suspicious for lung cancer, current guidelines now recommend histological confirmation through biopsy or surgical excision. As a result, the number of invasive biopsy procedures and surgical resections for early-stage disease has risen substantially^[^4,5^]^.

For over a decade, percutaneous transthoracic needle biopsy (PCNB) has been widely utilized for diagnosing lung cancer due to its relatively safe and minimally invasive nature with high diagnostic accuracy for lung cancer^[^6^]^. The common complications associated with PCNB include pneumothorax, hemoptysis, and pulmonary hemorrhage which are generally non-life threatening^[^7^]^. However, ongoing debates persist regarding other issues with PCNB, such as the reliability of detection results influenced by the operator’s skill and the potential for unintended tumor dissemination along the needle tract during the procedure although these remain subjects of ongoing debate. While some studies indicate no association between PCNB and recurrence in Stage I patient, a meta-analysis has reported an increased risk of pleural recurrence and decreased survival in Stage I patients following PCNB^[^8,9^]^. Thus, a clear conclusion has yet to be reached.

Despite the clear diagnostic advantages of PCNB, concerns regarding its potential association with postoperative recurrence remains unresolved. To address this debate, we investigated RFS in lung cancer patients who underwent either PCNB or intraoperative surgical biopsy prior to curative resection. Furthermore, we examined the interaction between biopsy methods and tumor characteristics, including location and morphology, to identify CT-based subgroups in which the diagnostic approach may influence postoperative outcomes.

This cohort/cross-sectional/case-control study has been reported in line with the STROCSS guidelines^[^10^]^.

## Materials and methods

The work has been reported in line with the STROCSS criteria^[^10^]^.

The study was approved by the Institutional Review Board of Korea University Anam Hospital (IRB No. 2025AN0124), and the requirement for individual informed consent was waived due to the retrospective nature of the study.

### Study population

This study is a retrospective review of medical records of patients who were diagnosed with primary lung cancer and received lung resection surgery at a single tertiary hospital between January 2015 and December 2020. Only patients who subsequently underwent curative resection after either PCNB or intraoperative surgical biopsy were included for analysis in order to reflect postoperative outcomes among resected lung cancer patients following different diagnostic pathways, rather than a direct comparison of diagnostic procedures themselves. Patients were excluded if they received neoadjuvant chemotherapy or radiotherapy, had double primary lung cancer, were under concurrent treatment for other malignancies, diagnosed with adenocarcinoma in situ or minimally invasive adenocarcinoma, or had insufficient medical records.

### Selection of biopsy method

At our institution, the choice between preoperative PCNB and intraoperative surgical biopsy was primarily determined through a multidisciplinary team (MDT) discussion involving thoracic surgeons, interventional radiologists, and pulmonologists. The MDT considered several key clinical and radiologic factors that influenced biopsy selection. PCNB was generally favored for peripheral lesions with a safe and direct needle trajectory, sufficient solid components to permit reliable sampling, or in patients with significant comorbidities or impaired pulmonary function where avoiding general anesthesia was desirable.

In contrast, intraoperative surgical biopsy was selected when PCNB was technically challenging or carried a higher procedural risk, such as in centrally located tumors, lesions adjacent to major vessels or bronchi, diffuse emphysematous lungs with high pneumothorax risk, dominant ground-glass nodules (GGN) with low PCNB yield expectancy. Surgical biopsy was also chosen in cases where a prior PCNB had been nondiagnostic despite persistent radiologic features suggestive of malignancy, necessitating definitive intraoperative tissue confirmation.

HIGHLIGHTS

- PCNB raises concerns of tumor dissemination and recurrence risk.
- Locoregional and pleural recurrences were more frequent in PCNB group.
- CTR and central tumor location predicted reduced recurrence-free survival.
- RFS decline was more pronounced in PCNB with high CTR or central tumors.
- Surgical biopsy or sub-lobar resection may be preferable in high-risk patients.

### Chest CT evaluation of tumor characteristics and location

Preoperative chest CT images were reviewed by two thoracic surgeons in collaboration with a board-certified thoracic radiologist who was blinded to the biopsy method and clinical outcomes. Tumor characteristics and location were analyzed on CT scans acquired with a slice thickness of 3 mm and reconstructed using a standard lung kernel. All lesions were evaluated on both lung and mediastinal window settings. Components visible on the mediastinal window were classified as solid (tumor), whereas components not visible were defined as ground-glass (consolidation).

The CTR was measured manually using electronic calipers. On the image demonstrating the largest tumor dimension, the maximal tumor diameter was recorded, after which the maximal diameter of the consolidation component was measured. CTR was calculated by dividing the maximal consolidation diameter by the maximal overall tumor diameter.

The consolidation to tumor ratio (CTR) was calculated for all patients and categorized into four groups (CTR group): CTR 0 (pure GGN), CTR 0.1–0.49, CTR 0.5–0.99, and CTR 1 (pure solid).

Tumor locations were classified as peripheral (outer one-third of the pulmonary lobe) or central (inner two-thirds). The distance of the tumor from the pleural surface was also measured in millimeters. Both the shallowest (closest) and deepest (farthest) perpendicular distances from the nearest pleura were measured and recorded.

Representative CT images demonstrating the CTR and intralobar tumor location is included as a Supplemental Digital Content Figure 1, available at: http://links.lww.com/JS9/H89.

**Figure 1.**
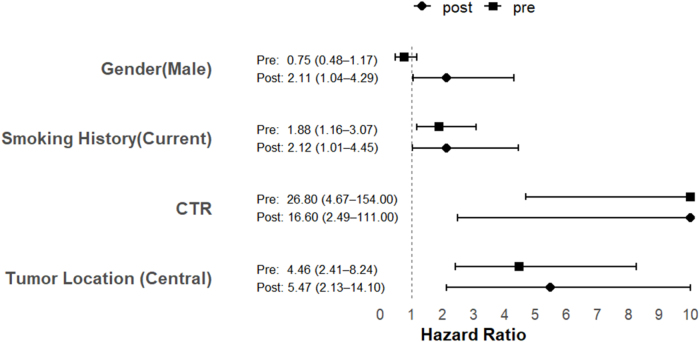
Forest plot demonstrating risk factors associated with recurrence in surgically resected lung cancer. Note. The numbers in the middle column represent hazard ratios for both biopsy methods both before (pre) and after (post) matching. The numbers in parenthesis represents 95% confidence interval range. RFS = Recurrence-free survival, PSM = Propensity score matching, CI = Confidence interval, CTR = consolidation-to-tumor ratio.

Lastly, the actual depth of the PCNB needle insertion was measured in millimeters and recorded using the CT image acquired during the procedure.

### PCNB procedures

The procedure was performed by experienced thoracic radiologists following the 2020 Korean Society of Thoracic Radiology guidelines^[^11^]^.

All PCNBs were performed under CT fluoroscopic guidance using 18-gauge core needles via a coaxial system. Two passes are were routinely made to obtain adequate tissue samples.

Patients with confirmed lung cancer underwent curative surgical resection. In cases where PCNB did not yield definitive histologic confirmation, intraoperative surgical biopsy was performed for diagnosis confirmation before proceeding with curative resection.

### Surgical procedure

All surgical procedures were conducted via video-assisted thoracic surgery (VATS). Patients without preoperative histological confirmation underwent intraoperative surgical biopsy through sub-lobar resections such as wedge resection or segmentectomy, followed by frozen-section analysis. The extent of final curative resection was determined based on tumor characteristics and location. Mediastinal lymph node dissection was performed in all lung cancer patients, with at least five stations resected, including lymph node stations 7, 9, 10, and 11.

### Post-operative follow-up and the definition of recurrence

Patients underwent postoperative follow-up for a minimum of five years and follow-up was conducted according to institutional postoperative surveillance protocols. Chest CT was routinely obtained at 1 month, 3 months, and 6 months after surgery, followed by imaging every 6 months for the first 2 years and annually thereafter. PET-CT or brain MRI was obtained when clinically indicated or when recurrence was suspected on routine CT. All suspected recurrences were reviewed in a MDT conference to reach a consensus diagnosis. Recurrences were categorized as locoregional (originating from the primary lobe, hilum, or surgical staple line, involving hilar, subcarinal, paratracheal lymph nodes, or other ipsilateral lobes), distant (other organs or contralateral lung), or multiple (simultaneous recurrences in multiple regions). Pleural recurrence was defined as ipsilateral pleural nodules, pleural seeding, or malignant effusion without evidence of distant metastasis.

Pleural recurrence was defined as recurrence in the parietal pleura adjacent to the original tumor location or in the pleura adjacent to the puncture site of a PCNB.

Tumor recurrence and second primary lung cancer were distinguished by considering factors such as disease-free interval, histologic type, radiological characteristics such as nodule morphology, location, lymph node enlargement, and PET-CT uptake. Lesions that differed histologically from the primary tumor or arose in a different lobe or contralateral lung after a disease-free interval of ≥2 years were regarded as second primary lung cancers and were therefore excluded from the recurrence analysis.

### Group comparison

The entire cohort of patients was divided into two groups: those who underwent PCNB and those who underwent intraoperative biopsy (Surgical biopsy) for comparative analysis. Clinical data such as gender, age, radiological features, prior disease history, smoking history, pathological findings, recurrence data, death, and other treatment-related details were collected for analysis. Pathological stages were classified in accordance with the eighth edition of the TNM International Staging System.

### Statistical analysis

All data were recorded using Microsoft Excel (Microsoft Corp., Bellevue, WA). Baseline characteristics are presented as mean ± standard deviation (SD) or median with range [min-max] for continuous variables, and as number (percentage) for categorical variables. The mean with SD was used for normally distributed data without significant outliers, whereas the median with range was applied to variables with skewed distributions or notable outliers. Categorical variables were analyzed using the Chi-square test or Fisher’s exact test, as appropriate, while continuous variables were assessed using the Mann–Whitney U-test.

Logistic regression was used to calculate estimates for propensity score matching (PSM). The independent variables considered for matching were age, gender, CTR, CTR Group, tumor location, histologic type, tumor invasiveness, and smoking status. Propensity scores were estimated by logistic regression and matched in a 1:1 nearest-neighbor fashion (caliper 0.2, no replacement). Covariate balance was evaluated using standardized mean differences (SMD), with >90 % of variables achieving SMD < 0.1. A sensitivity analysis using inverse probability of treatment weighting (IPTW) produced similar results.

For the analysis of risk factors for RFS, we employed multivariable Cox regression analysis. Adjusted hazard ratios (HR) were calculated using the variables considered for matching as covariates. Variables entered into the multivariable Cox regression model were selected based on clinical relevance and statistical significance in univariable analyses (*P* < 0.1). The proportional hazards assumption was evaluated using the Grambsch and Therneau test based on Schoenfeld residuals and no violation was observed for the key variables, including PCNB. Unadjusted HRs were also calculated using multivariable Cox regression, treating each matched dataset as a cluster to account for the clustering effect. As a sensitivity analysis, we performed an IPTW analysis using stabilized weights to further adjust for baseline differences between groups. The weighted Cox model yielded results that were consistent with those of the primary propensity-matched analysis. This result is shown in Supplementary Digital Content Table 1, available at: http://links.lww.com/JS9/H90.

The primary endpoint was RFS and it was estimated using the Kaplan–Meier method, and a *P* < 0.05 was considered statistically significant.

Statistical analysis was performed using R, version 4.5.1 (R Foundation for Statistical Computing, Vienna, Austria), and Python (https://www.python.org/, Version 3.10.12, Python Software Foundation, Wilmington, DE, USA) employing libraries such as StatsModels, SciPy, Pandas, NumPy, and Scikit-learn for statistical modeling and analysis.

All statistical analyses, including propensity-score estimation and Cox regression modeling, were conducted under the supervision of a certified statistician to ensure methodological validity and reproducibility.

## Results

A total of 363 patients were diagnosed with primary lung cancer and underwent thoracoscopic surgery. Among these, 142 patients underwent surgical biopsy without prior PCNB, while 221 patients received preoperative PCNB. The clinical demographics of the enrolled patients, both before and after matching, are summarized in Table 1 and Table 2, respectively.

**Table 1:** Baseline characteristics of biopsy groups before propensity score matching.

Variable	Surgical biopsy (*N*= 142)	PCNB (*N* = 221)	*P*	SMD
Age (years)	66 [58.25–72]	67 [61–73]	0.17	0.14
Gender, *n* (%)			0.55	0.08
Male	78 (54.9)	113 (51.1)		
Female	64 (45.1)	108 (48.9)		
BMI (kg/m^2^)	24.3 [22.3–26.1]	24.6 [22.9–26.2]	0.55	0.004
Smoking history, *n* (%)			0.73	0.09
Never	79 (55.6)	123 (55.7)		
Current	32 (22.5)	56 (25.3)		
Ex-smoker	31 (21.8)	42 (19)		
Prior cancer, *n* (%)	41 (28.9)	41 (18.6)	0.050	0.24
Median follow-up (days)	2179 [1746–2779]	1979 [1478–2626]	0.38	0.21
Tumor size, (mm)	15 [7.3–21]	22 [15–31]	<0.001	0.70
Consolidation size (mm)	19 [15–26.8]	25 [20–35]	<0.001	0.56
CTR	0.74 [0.43–1]	0.96 [0.71–1]	<0.001	0.62
CTR group, *n* (%)			<0.001	0.64
Pure GGN	10 (7)	1 (0.5)		
0.1–0.49	31 (21.8)	13 (5.9)		
0.5–0.99	53 (37.3)	97 (43.9)		
Pure solid	48 (33.8)	110 (49.8)		
Tumor location, *n* (%)			0.11	0.19
Peripheral	67 (47.2)	84 (38)		
Central	75 (52.8)	137 (62)		
Tumor depth shallow (mm)	2.4 [0.0–9.8]	0.0 [0.0–5.0]	0.039	0.42
Tumor depth deepest (mm)	23.3 [16.7–31.3]	27.5 [19.5–35.7]	0.010	0.37
PCNB needle depth (mm)	N/A	34.4 [26.7–43.9]	< 0.001	3.82
Histology type, *n* (%)			0.79	0.08
ADC	114 (80.3)	171 (77.4)		
SqCC	23 (16.2)	42 (19)		
Other	5 (3.5)	8 (3.6)		
VPI, *n* (%)	73 (51.4)	213 (70.1)	<0.001	0.39
Pathologic stage, *n* (%)			0.005	0.42
IA	55 (38.7)	46 (20.8)		
IB	70 (49.3	132 (59.7)		
IIA	5 (3.5)	12 (5.4)		
IIB	12 (8.5)	30 (13.6)		
IIIA	0 (0)	1 (0.5)		
Pathologic T stage, *n* (%)			0.003	0.50
T1a	21 (14.8)	15 (6.8)		
T1b	28 (19.7)	27 (12.2)		
T1c	12 (8.5)	10 (4.5)		
T2a	75 (52.8)	139 (62.9)		
T2b	3 (2.1)	13 (5.9)		
T3	3 (2.1)	16 (7.2)		
T4	0 (0)	1 (0.5)		
Pathologic N stage, *n* (%)			0.049	0.42
N0	131 (92.3)	208 (94.1)		
N1	11 (7.7)	13 (5.9)		
OP extent, *n* (%)			<0.001	0.53
Wedge	25 (17.6)	10 (4.5)		
Segmentectomy	9 (6.3)	3 (1.4)		
Lobectomy	108 (76.1)	208 (94.1)		
OP duration (min)	175 [140–210]	185 [145–240]	0.050	0.35
Recurrence, *n* (%)	22 (15.5)	60 (27.1)	0.050	0.29
Recurrence site, *n* (%)			0.48	0.37
Locoregional	11 (50.0)	35 (58.3)		
Distant	4 (18.2)	15 (25.0)		
Multiple	7 (31.8)	10 (16.7)		
Pleural recurrence, *n* (%)	2 (1.4)	11 (5)	0.14	0.20
Death, *n* (%)	15 (10.6)	24 (10.9)	>0.99	0.01
30-day Complication, *n* (%)	13 (9.2)	37 (16.7)	0.060	0.23
Recurrence since OP (days)	546 [403–859]	797 [521–1130]	0.16	0.35
Death since OP (days)	854 [579–1743]	1446 [1145–1824]	0.23	0.42

Note. Continuous variables presented as the mean ± SD or median [range], categorical variables presented as no. (%)

PCNB = Percutaneous needle biopsy, SMD = Standardized mean difference, BMI = Body-Mass Index, CTR = Consolidation to Tumor Ratio, GGN = Ground-Glass Nodule, ADC = Adenocarcinoma, SqCC = Squamous cell carcinoma, VPI = Visceral Pleura Invasion.

**Table 2:** Baseline characteristics of biopsy groups after propensity score matching.

Variable	Surgical biopsy(*N* = 103)	PCNB(*N* = 103)	*P*	SMD
Age (years)	66 [59–72]	69 [59–73]	0.18	0.17
Gender, *n* (%)			0.67	0.08
Male	51 (51.0)	55 (55.0)		
Female	49 (49.0)	45 (45.0)		
BMI (kg/m^2)^	24.3 [22.3–26.0]	24.1 [22.5–25.6]	0.71	0.06
Smoking history, *n* (%)			0.91	0.06
Never	56 (56.0)	56 (56.0)		
Current	25 (25.0)	23 (23.0)		
Ex-smoker	19 (19.0)	21 (21.0)		
Prior cancer, *n* (%)	27 (27.0)	20 (20.0)	0.32	0.17
Total follow-up (days)	1120 [801–1580]	1060 [800–1495]	0.21	0.13
Tumor size (mm)	16.0 [10.5–23.5]	19.0 [13.0–26.5]	0.050	0.30
Consolidation size (mm)	21.0 [16.0–28.5]	24.0 [19.0–32.0]	0.04	0.40
CTR	0.83 [0.56–1.00]	0.85 [0.63–1.00]	0.46	0.03
CTR Group, *n* (%)			>0.99	0.02
Pure GGN	1 (1.0)	1 (1.0)		
0.1–0.49	13 (13.0)	13 (13.0)		
0.5–0.99	48 (48.0)	49 (49.0)		
Pure solid	38 (38.0)	37 (37.0)		
Tumor location, *n* (%)			0.67	0.08
Peripheral	55 (55.0)	59 (59.0)		
Central	45 (45.0)	41 (41.0)		
Tumor depth shallow (mm)	2.4 [0.0–9.8]	0.0 [0.9–5.6]	0.070	0.25
Tumor depth deepest (mm)	23.3 [16.7–31.3]	27.6 [20.6–35.6]	<0.001	0.39
PCNB needle depth (mm)	N/A	35.5 [27.3–44.5]	<0.001	3.93
Histology type, *n* (%)			>0.99	0.00
ADC	77 (77.0)	77 (77.0)		
SqCC	19 (19.0)	19 (19.0)		
Other	4 (4.0)	4 (4.0)		
VPI, *n* (%)	54 (54.0)	66 (66.0)	0.11	0.25
Pathologic stage, *n* (%)			0.62	0.19
IA	35 (35.0)	27 (27.0)		
IB	49 (49.0)	57 (57.0)		
IIA	5 (5.0)	4 (4.0)		
IIB	11 (11.0)	12 (12.0)		
Pathologic T stage, *n* (%)			0.54	0.29
T1a	110	10 (10.0)		
T1b	120	20 (20.0)		
T1c	130	9 (9.0)		
T2a	210	55 (55.0)		
T2b	220	3 (3.0)		
T3	300	3 (3.0)		
Pathologic N stage, *n* (%)			0.052	0.32
N0	89 (89.0)	97 (97.0)		
N1	11 (11.0)	3 (3.0)		
OP extent, *n* (%)			0.90	0.06
Wedge	9 (9.0)	9 (9.0)		
Segmentectomy	2 (2.0)	3 (3.0)		
Lobectomy	89 (89.0)	88 (88.0)		
OP duration (min)	185 [155–215]	180 [145–217]	0.47	0.25
Recurrence, *n* (%)	14 (14.0)	22 (22.0)	0.20	0.21
Recurrence site, *n* (%)			0.22	0.51
Locoregional	8 (57.1)	16 (72.7)		
Distant	2 (14.3)	4 (18.2)		
Multiple	4 (28.6)	2 (9.1)		
Pleural recurrence, *n* (%)	1 (1.0)	2 (2.0)	> 0.99	0.08
Death, *n* (%)	10 (10.0)	6 (6.0)	0.43	0.15
30-day Complication, *n* (%)	10 (10.0)	13 (13.0)	0.66	0.09
Recurrence since OP (days)	1080 [700–1520]	1010 [630–1390]	0.14	0.33
Death since OP (days)	1840 [1410–2260]	1810 [1370–2190]	0.30	0.84

Note. Continuous variables presented as the mean ± SD or median [range], categorical variables presented as no. (%)

PCNB = Percutaneous needle biopsy, SMD = Standardized mean difference, BMI = Body-Mass Index, CTR = Consolidation-to-Tumor Ratio, GGN = Ground-Glass Nodule, ADC = Adenocarcinoma, SqCC = Squamous cell carcinoma, VPI = Visceral Pleura Invasion.

According to the pre-PSM data presented in Table 1, recurrence was significantly more frequent in the PCNB group compared with the surgical biopsy group (27.1% vs. 15.5%, *P* = 0.050). When stratified by recurrence site, the PCNB group consistently showed a higher number of patients with locoregional recurrence (11 vs. 35), distant metastasis (4 vs. 15), and multiple metastases (7 vs. 10) but without statistical significance (*P* = 0.48). The incidence of pleural metastasis, regarded as a critical complication of PCNB, was notably higher in the PCNB group compared with the surgical biopsy group (2 vs. 11, *P* = 0.14).

After PSM, 103 patients were included in each group, as shown in Table 2. The PCNB group had larger tumor sizes (19 mm vs. 16 mm, *P* = 0.050), larger consolidation sizes (24 mm vs. 21 mm, *P* = 0.040), and greater tumor depth from the visceral pleura (27.6 mm vs. 23.3 mm, *P* < 0.001). However, there were no significant differences between the two groups in terms of the number of recurrences (22 vs. 14, *P* = 0.20), recurrence site (*P* = 0.20), or pleural recurrence (2 vs. 1, *P* = 0.22). All other baseline characteristics were generally well balanced between the two groups following PSM.

There was no significant difference in 30-day postoperative complications between the two groups, both before and after PSM. No major complications were observed, and the most common complication was prolonged air leakage, which did not require any additional intervention before discharge.

Table 3 and Figure 1 present the key risk factors associated with RFS, both before and after PSM. After PSM, male gender (HR 2.11, *P* = 0.040), current smoking status (HR 2.12, *P* = 0.048), a centrally located tumor (HR 5.47, *P* < 0.001), and an increased CTR (HR 16.6, *P* = 0.004) were found to be significantly associated with RFS. In contrast, PCNB was not identified as a statistically significant risk factor for recurrence (HR, 1.62, *P* = 0.16), and neither was visceral pleural invasion (VPI) (HR 1.80, *P* = 0.11).

**Table 3:** Multivariable Cox regression analysis for recurrence risk before and after propensity score matching.

Event	Before matching		After matching	
	Unadjusted HR (95% CI)	*P*	Adjusted HR(95% CI)	*P*
Age	1.02 (0.99–1.04)	0.16	1.03 (1.00–1.07)	0.070
Gender				
Male	0.75 (0.48–1.17)	0.21	2.11 (1.04–4.29)	0.040
Smoking history				
Never	(Reference)		(Reference)	
Current	1.88 (1.16–3.07)	0.010	2.12 (1.01–4.45)	0.048
PCNB	1.32 (0.81–2.16)	0.26	1.62 (0.83–3.16)	0.16
CTR	26.8 (4.67–154.0)	< 0.001	16.6 (2.49–111.0)	0.004
Tumor location				
Peripheral	(Reference)		(Reference)	
Central	4.46 (2.41–8.24)	< 0.001	5.47 (2.13–14.1)	< 0.001
VPI	1.62 (0.98–2.68)	0.060	1.80 (0.87–3.74)	0.11
OP Extent				
Wedge	(Reference)		(Reference)	
Segmentectomy	0.20 (0.03–1.58)	0.13	1.86 (0.17–20.50)	0.61
Lobectomy	0.51 (0.27–0.97)	0.021	1.61 (0.39–6.70)	0.52

Note. Numbers on the left represent the hazard ratios and the numbers in the parentheses indicate the 95% confidence intervals.

HR = Hazard ratio, CI = Confidence interval, PCNB = Percutaneous needle biopsy, CTR = Consolidation-to-Tumor Ratio, VPI = Visceral Pleura Invasion.

**Figure 2.**
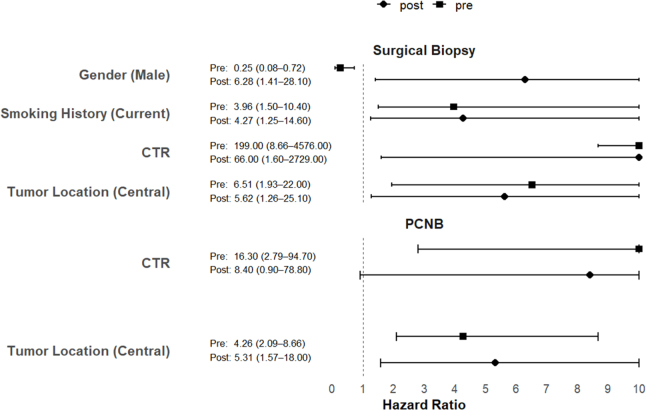
Impact of biopsy technique on recurrence: hazard ratios before and after propensity score matching. Note. The numbers in the middle column represent hazard ratios for both biopsy methods both before (pre) and after (post) matching. The numbers in parenthesis represents 95% confidence interval range. PCNB = Percutaneous needle biopsy, RFS = Recurrence-free survival, PSM = Propensity score matching, CI = Confidence interval, CTR = consolidation-to-tumor ratio.

Impact of surgical biopsy and PCNB on RFS are shown in Table 4 and Figure 2. Within the surgical biopsy group, male gender (HR 6.28, *P* = 0.020), current smoking history (HR 4.27, *P* = 0.020), increased CTR (HR 66, *P* = 0.030), and centrally located tumors (HR 5.62, *P* = 0.030) were identified as risk factors for recurrence. In contrast, increased CTR (HR 8.40, *P* = 0.040) and centrally located tumors (HR 5.31, *P* = 0.007) were identified as significant risk factors for recurrence within the PCNB group.

**Table 4:** Multivariable Cox regression analysis of recurrence risk stratified by biopsy method.

Event	Surgical Biopsy		PCNB	
	Adjusted HR(95% CI)	*P*	Adjusted HR(95% CI)	*P*
Age	1.01 (0.95–1.07)	0.75	1.05 (1.00–1.10)	0.055
Gender				
Male	6.28 (1.41–28.1)	0.020	1.15 (0.49–2.70)	0.74
Smoking history				
Never	(Reference)		(Reference)	
Current	4.27 (1.25–14.6)	0.020	1.27 (0.47–3.44)	0.64
CTR	66.00 (1.60–2729.0)	0.030	8.40 (0.90–78.8)	0.040
Tumor location				
Peripheral	(Reference)		(Reference)	
Central	5.62 (1.26–25.1)	0.030	5.31 (1.57–18.0)	0.007
VPI	2.26 (0.71–7.22)	0.090	1.38 (0.54–3.53)	0.51

Note. Numbers on the left represent the hazard ratios and the numbers in the parentheses indicate the 95% confidence intervals

RFS = Recurrence-free survival, PSM = Propensity score matching, CI = Confidence interval, PCNB = Percutaneous needle biopsy, CTR = Consolidation-to-Tumor Ratio, VPI = Visceral Pleura Invasion.

**Figure 3.**
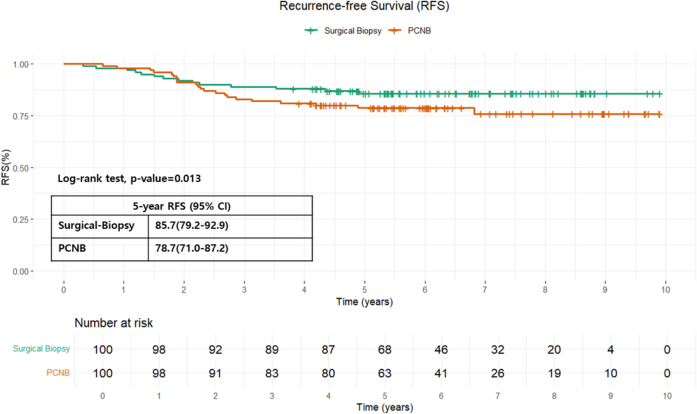
Kaplan–Meier analysis of recurrence-free survival by biopsy method after propensity score matching. RFS = Recurrence-free survival, PSM = Propensity score matching, CI = Confidence interval, PCNB = Percutaneous needle biopsy, CTR = consolidation-to-tumor ratio.

**Figure 4.**
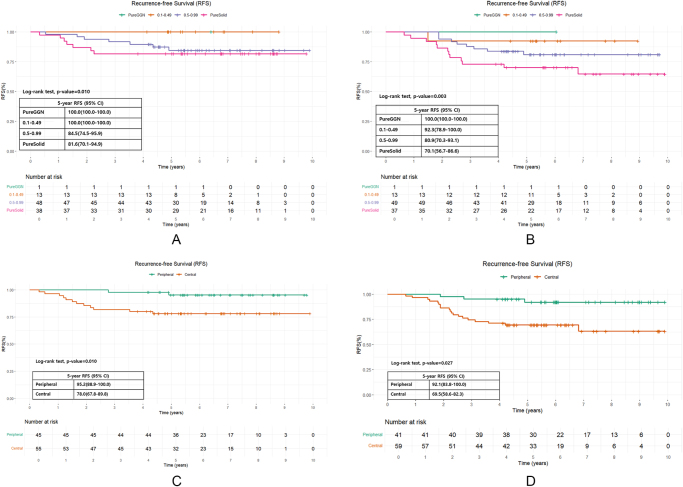
(A) RFS by CTR group (Surgical biopsy), (B) RFS by CTR group (PCNB), (C) RFS by Tumor locations (Surgical biopsy), (D) RFS by Tumor location (PCNB). RFS = Recurrence-free survival, CI = Confidence interval, PCNB = Percutaneous needle biopsy, CTR = consolidation-to-tumor ratio.

Figure 3 presents the Kaplan–Meier curves comparing the 5-year RFS between the surgical biopsy and PCNB groups. Figure 4 illustrates the RFS curves, stratified by biopsy method, for subgroups based on CTR group and tumor location. According to Figure 3, a significant difference in 5-year RFS was observed between the surgical biopsy and PCNB group (85% vs. 74.3%, *P* = 0.013).

RFS stratified by CTR group and tumor location is shown in Figure 4. Figure 4A and B depict the RFS of each biopsy group according to CTR group. In both plots, an increasing CTR was associated with a decline in 5-year RFS, with the PCNB group consistently showing lower RFS compared with the surgical biopsy group. Similarly, Figure 4C and D, which illustrate RFS according to tumor location, demonstrate that the PCNB group exhibited reduced 5-year RFS for both peripheral and central tumors.

## Discussion

The increasing detection rate of early-stage pulmonary nodules highly suspicious for lung cancer represents an important advance in thoracic oncology. Since histologic confirmation remains essential for definitive diagnosis of lung cancer, clinicians must choose between preoperative tissue confirmation using less invasive modalities such as PCNB versus proceeding directly to surgical resection, which allows simultaneous histological confirmation and curative intent treatment. CNB has long been accepted as a reliable and relatively safe diagnostic modality, given its minimally invasive nature and low incidence of serious complications^[^6,7,12^]^. However, as the procedure inherently involves traversing the tumor and withdrawing the needle, concerns have been raised regarding the potential for tumor cell displacement along the needle tract, which could theoretically contribute to recurrence^[^6,8,9^]^.

Tumor cell implantation during PCNB is thought to occur when viable tumor cells are dislodged and carried along the needle tract during withdrawal, subsequently seeding adjacent soft tissue or the pleural surface^[^13–15^]^. Several studies have identified factors that may increase susceptibility to such implantation, including histologic types such as adenocarcinoma or small cell carcinoma, the presence of spread through air spaces (STAS), the use of larger-gauge or non-coaxial needles, and multiple PCNB attempts^[^15–18^]^. Subpleural tumors have also been associated with an increased risk of pleural recurrence, likely due to minimal tissue barriers between the lesion and pleural surface^[^19^]^. Although these mechanisms provide a theoretical basis for tumor dissemination, reported incidence rates vary widely across studies, and the clinical significance remains uncertain.

In a large-scale survey conducted by Tomiyama et al., needle-tract seeding was reported in 6 out of 9783 PCNB cases across 124 centers in Japan, corresponding to an incidence of 0.061%. Similarly Komiya *et al* observed cancer cell implantation along the needle biopsy tract in only 1 of 420 patients who underwent PCNB^[^20,21^]^. In contrast, a study published by Kim et al. in 2022 reported a pleural recurrence rate of 9.6% (40 out of 415) in Stage I lung cancer patients who underwent PCNB^[^22^]^. Furthermore, a meta-analysis published in 2021 reported that PCNB increased the risk of ipsilateral pleural recurrence and was associated with reduced survival in patients younger than 55 years with Stage I lung cancer^[^9^]^. Subsequent recent studies have also identified PCNB as a risk factor for recurrence^[^6,23,24^]^.

When interpreted alongside this heterogeneous body of literature, our findings help clarify potential reasons for these discrepant results. In our study, PCNB was not an independent predictor of recurrence after adjusting for confounders however, pleural and other locoregional recurrences occurred numerically more often in the PCNB group. Importantly, these differences appeared to be mediated largely by underlying radiologic tumor morphology, specifically tumors with higher CTR and central tumor location, both of which were strong independent predictors of recurrence. Many earlier studies did not adjust for imaging-based invasive features such as CTR or solidity, which may have inflated the apparent effect attributed to PCNB itself. Factors related to procedural techniques may also contribute to inconsistent findings in prior literature. Variations in needle gauge, use of a coaxial system, biopsy trajectory relative to the solid component, the number of needle passes, and operator experience may further contribute to inconsistencies across prior reports.

Although our current study did not exclusively target early-stage lung cancer, all analysis was conducted after controlling selection bias between the surgical biopsy and PCNB groups through PSM, which revealed several key insights. First, we found that PCNB showed a non-significant trend toward increased recurrence risk both before (HR 1.32, 95% CI 0.81–2.16, *P* = 0.26) and after matching (HR 1.62, 95% CI 0.83–3.16, *P* = 0.16) as shown in Table 3. Although these findings did not reach statistical significance, the consistent trend of hazard ratios and the higher rate of locoregional and pleural recurrence in the PCNB group raise the possibility of clinically meaningful effects that should be interpreted cautiously and not in a strictly binary statistical framework. Given the modest number of events, limited statistical power may partly explain the non-significant results.

To further clarify this result, we performed a post-hoc power analysis. A post-hoc power analysis indicated that, given this number of events, only large effects (detectable HR = 2.4 at 80 % power) could be statistically detected. Since the observed HR for PCNB was 1.62, the study was likely underpowered to identify such a modest difference as significant. This result suggests that the lack of statistical significance is mainly due to the small size of the effect rather than a true absence of difference.

Second, both CTR and a central tumor location emerged as strong predictors of recurrence independent of biopsy method. This finding aligns with the current NCCN guidelines, which emphasize radiologic assessment of lesion solidity and location when selecting biopsy techniques^[^4^]^. These findings emphasizes an importance of incorporating high risk CT characteristics and support the concept of a tailored biopsy strategy.

Finally, as shown in Figure 4, declines in RFS among the PCNB group became more pronounced with increasing CTR and central location. Although these patterns did not reach statistical significance, the decline in the PCNB group became more pronounced as tumor solidity increased.

Moreover, RFS was significantly reduced for centrally located tumors in both groups, with a notably steeper decline observed in the PCNB cohort. While CTR and tumor location are well-established prognostic factors, our findings suggest that their adverse influence on recurrence may vary depending on the diagnostic pathway used. These patterns should be viewed as preliminary signals of a potential interaction between tumor morphology and biopsy approach, underscoring the need for prospective studies employing standardized PCNB techniques to clarify these relationships.

This study has several limitations. First, it is a retrospective analysis conducted at a single institution, which may limit the generalizability of the findings. Although the initial cohort consisted of 363 patients, only 103 patients remained in each group after PSM, which may have limited the statistical power for multivariate and subgroup analyses. Second, because the biopsy method was determined through MDT discussion, decisions were inevitably influenced by temporal practice patterns and case-specific clinical considerations, rendering some degree of selection bias unavoidable. These factors were mitigated through PSM and multivariate adjustment but, residual confounding cannot be fully excluded. Third, the assessment of CTR and tumor location from preoperative CT scans may have been subject to interobserver bias. One notable limitation of CTR calculation during the study period was the difficulty in accurately delineating the boundary between the consolidation and the tumor, particularly in cases with lower tumor solidity. This difficulty introduces potential interobserver variability that may affect the consistency and reliability of CTR-based stratification. Although inter-observer agreement was not quantitatively assessed, the measurements were reviewed in consensus among the three experts to ensure reliability and consistency. Furthermore, CTR and location were visually assessed rather than utilizing AI-based quantification. Future automated approaches may improve reproducibility.

Another limitation is that a competing-risk model could not be performed because the number of distant metastasis events was insufficient to yield statistically reliable estimates.

Lastly, histologic factors such as spread through air spaces (STAS) were not routinely reported during our study period and thus not included in the multivariate analysis. Their absence may represent an unmeasured confounder.

Despite these limitations, our study has notable strengths. To our knowledge, this is the first study to incorporate CTR and intralobar tumor location as CT findings to analyze the RFS of patients who underwent either surgical biopsy or PCNB. In the current clinical environment, where CT-based lung cancer screening is widely implemented, the early detection rate of small-sized or subsolid lung tumors has increased substantially, rendering the CTR highly significant in clinical practice. CTR provides essential clinical information for guiding follow-up strategies and predicting patient prognosis^[^25,26^]^. In addition, CTR is a important factor in surgical decision-making, particularly in determining tumor location and the extent of resection, such as wedge resection or segmentectomy^[^27,28^]^. Therefore, in patients with CT features that suggest a higher recurrence risk, it may be reasonable to consider upfront surgical biopsy or sub-lobar resection which offers the advantage of achieving both histological diagnosis and curative resection in a single procedure. Nonetheless, these considerations should be individualized, and further evidence is needed to refine optimal biopsy strategies in this subset of patients.

## Conclusion

In conclusion, PCNB itself was not an independent determinant of recurrence. Nevertheless, its association with higher number of locoregional and pleural recurrence among tumors with high CTR or central location highlights the importance of integrating preoperative imaging morphology into diagnostic planning. Lung cancers these high-risk radiologic features may warrant a more cautious and individualized approach when selecting a method for histologic confirmation. For these high-risk morphologic subgroups, surgical biopsy or sub-lobar resection may be considered for simultaneous histological confirmation and curative resection. While PCNB was not an independent risk factor for recurrence, its association with locoregional and pleural events in high-CTR or central tumors warrants further prospective validation.

## Data Availability

The datasets used and/or analyzed during the current study are available from the corresponding author on reasonable request.

## Research registration unique identification number (UIN)

Research Registry: 11511 https://researchregistry.knack.com/researchregistry#home/registrationdetails/68bb8a4119959402b439318b/
